# A multilevel investigation to reveal the regulatory mechanism of lignin accumulation in juice sac granulation of pomelo

**DOI:** 10.1186/s12870-024-05095-4

**Published:** 2024-05-11

**Authors:** Luning Liu, Yiran Chen, Weilin Wu, Qiuyou Chen, Zhijiao Tian, Jiakang Huang, Huaqing Ren, Jiacheng Zhang, Xi Du, Mulai Zhuang, Ping Wang

**Affiliations:** 1 Insititute of Genetics and Breeding in Horticultural Plants, College of Horticulture, Fujian Agriculture and Forestry University, Fuzhou, China; 2 Bureau of Agriculture and Rural Affairs of Pinghe County, Pinghe, China

**Keywords:** Antioxidant enzyme, H_2_O_2_, Juice sac granulation, Lignin, Pomelo, ROS, Transcriptome, Ubiquitinome

## Abstract

Granulation of juice sacs is a physiological disorder, which affects pomelo fruit quality. Here, the transcriptome and ubiquitinome of the granulated juice sacs were analyzed in Guanxi pomelo. We found that lignin accumulation in the granulated juice sacs was regulated at transcription and protein modification levels. In transcriptome data, we found that the genes in lignin biosynthesis pathway and antioxidant enzyme system of the granulated juice sacs were significantly upregulated. However, in ubiquitinome data, we found that ubiquitinated antioxidant enzymes increased in abundance but the enzyme activities decreased after the modification, which gave rise to reactive oxygen species (ROS) contents in granulated juice sacs. This finding suggests that ubiquitination level of the antioxidant enzymes is negatively correlated with the enzyme activities. Increased H_2_O_2_ is considered to be a signaling molecule to activate the key gene expressions in lignin biosynthesis pathway, which leads to the lignification in granulated juice sacs of pomelo. This regulatory mechanism in juice sac granulation of pomelo was further confirmed through the verification experiment using tissue culture by adding H_2_O_2_ or dimethylthiourea (DMTU). Our findings suggest that scavenging H_2_O_2_ and other ROS are important for reducing lignin accumulation, alleviating juice sac granulation and improving pomelo fruit quality.

**Supplementary Information:**

The online version contains supplementary material available at 10.1186/s12870-024-05095-4.

## Background

Pomelo is a citrus fruit, native to China and now widely grown around the world, with high nutritional value [1]. Juice sac granulation, one of the serious physiological disorders in citrus fruits, occurs during harvest and postharvest storage of the fruits. The occurrence of pomelo juice sac granulation degrades fruit quality [2].

Recently, there have been several transcriptome researches regard to the juice sac granulation of citrus. Yao et al. [3] reported that the changes in gene expressions based on the transcriptome datasets in the metabolism of sugar and organic acid might be related to juice sac granulation by modulating cell wall components in postharvest Ponkan (*Citrus reticulata*). Kang et al. [4] indicated that the accumulated cell wall components such as lignin, cellulose and protopectins were closely related to the juice sac granulation by comparing the transcriptome profiles and physiological properties in different juice sacs of Huyou fruit (*Citrus changshanensis*). Furthermore, previous studies indicated that ROS and cell wall compositions are important intracellular factors affecting juice sac granulation of citrus fruit [5, 6]. ROS play a role in plant growth and development, acting both as crucial signal transduction molecules and on the other as toxic by-products that accumulate in cells under various stresses [7]. Superoxide dismutase (SOD), catalase (CAT), glutathione-S-transferase (GST) and glutathione peroxidase (GPX) are antioxidant enzymes acting as ROS scavengers. It has been reported that excessive ROS is scavenged via increasing the activities of SOD and CAT in postharvest Majia pomelo fruits coated with chitosan, which delays the juice sac granulation [8]. Moreover, the increased cell wall components such as lignin, pectin, and cellulose are closely associated with juice sac granulation of citrus [9]. Among them, the lignin accumulation is considered to be the most important factor causing juice sac granulation [10].

Ubiquitination is a crucial post-translational modification (PTM) of protein, which is important for regulating protein localization, functions and interactions in biological cells [11]. Previous proteomic investigations indicate that tens-of-thousands of ubiquitination sites on thousands of proteins are identified. It appears that most proteins will be ubiquitinated at some point in their cellular lifetime [12]. The increasing evidence shows that protein ubiquitination involves virtually all cellular processes and almost all events in the entire life cycle of plants [13]. Lu et al. [14] reported the ubiquitinome were changed in senescing rose petals, suggesting that ubiquitinated proteins played important roles in the metabolisms of the petals during senescence. He et al. [15] found that protein ubiquitination played a widely regulating role in rice seed germination by analyzing the ubiquitinome profiles. Furthermore, Mo et al. [16] reported that numerous enzymes such as sugar metabolism-related enzymes, 1-aminocyclopropane-1-carboxylic acid oxidases, endochitinase and cell wall invertase were identified as DUPs, which significantly changed during papaya ripening process. Fan et al. [17] highlighted that multiple cellular processes and diverse interactions were regulated by ubiquitinated proteins in the maize kernel development. However, protein ubiquitination related to fruit juice sac granulation has not been reported. Although many transcriptome studies have been carried out in granulated juice sacs of citrus fruits, we are not very clear about the molecular regulation of ubiquitinated proteins regarding to fruit juice sac granulation. Therefore, it is necessary to screen genes and ubiquitinated proteins on a large scale by analyzing transcriptome and ubiquitinome profiles in order to reveal the molecular mechanism of the juice sac granulation in pomelo.

## Results

### Phenotypic characteristics and lignin content in granulated juice sacs of pomelo

**Fig. 1:**
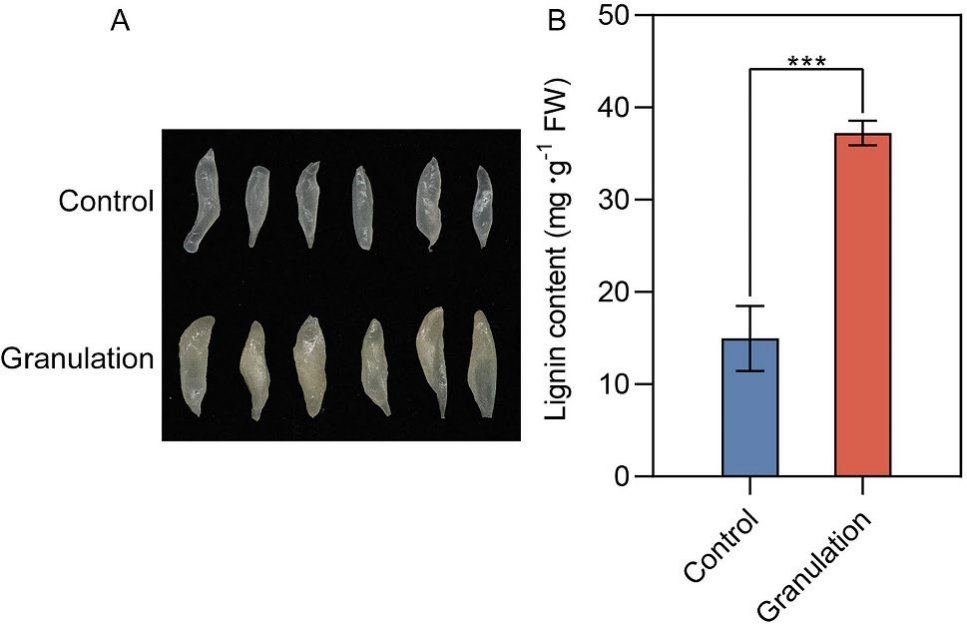
Changes of phenotype characteristics and lignin content of pomelo granulated juice sacs. Phenotype characteristics of normal and granulated juice sacs (**A**), lignin content (**B**) were shown. Control, normal juice sacs; Granulation, granulated juice sacs. The asterisks represented a significant difference at *p* < 0.05 level by Student’s *t*-test

Pomelo juice sac granulation obviously influences fruit quality. The results of the observation showed that the phenotypic characteristics of pomelo granulated juice sacs were markedly different from those of normal juice sacs in pomelo (Fig. 1A). The normal juice sacs were transparent and it was possible for the juice sacs to spill out after being cut. However, the granulated juice sacs became rough, hard, cloudy white or yellow. In addition, the lignin content was significantly increased by 2.48 times in the granulated juice sacs compared with normal ones (Fig. 1B). These results indicate that the phenotype characteristics and quality of juice sacs has been changed in the granulated pomelo.

### Identification and functional annotation of differentially expressed genes (DEGs) in granulated juice sacs of pomelo

**Fig. 2:**
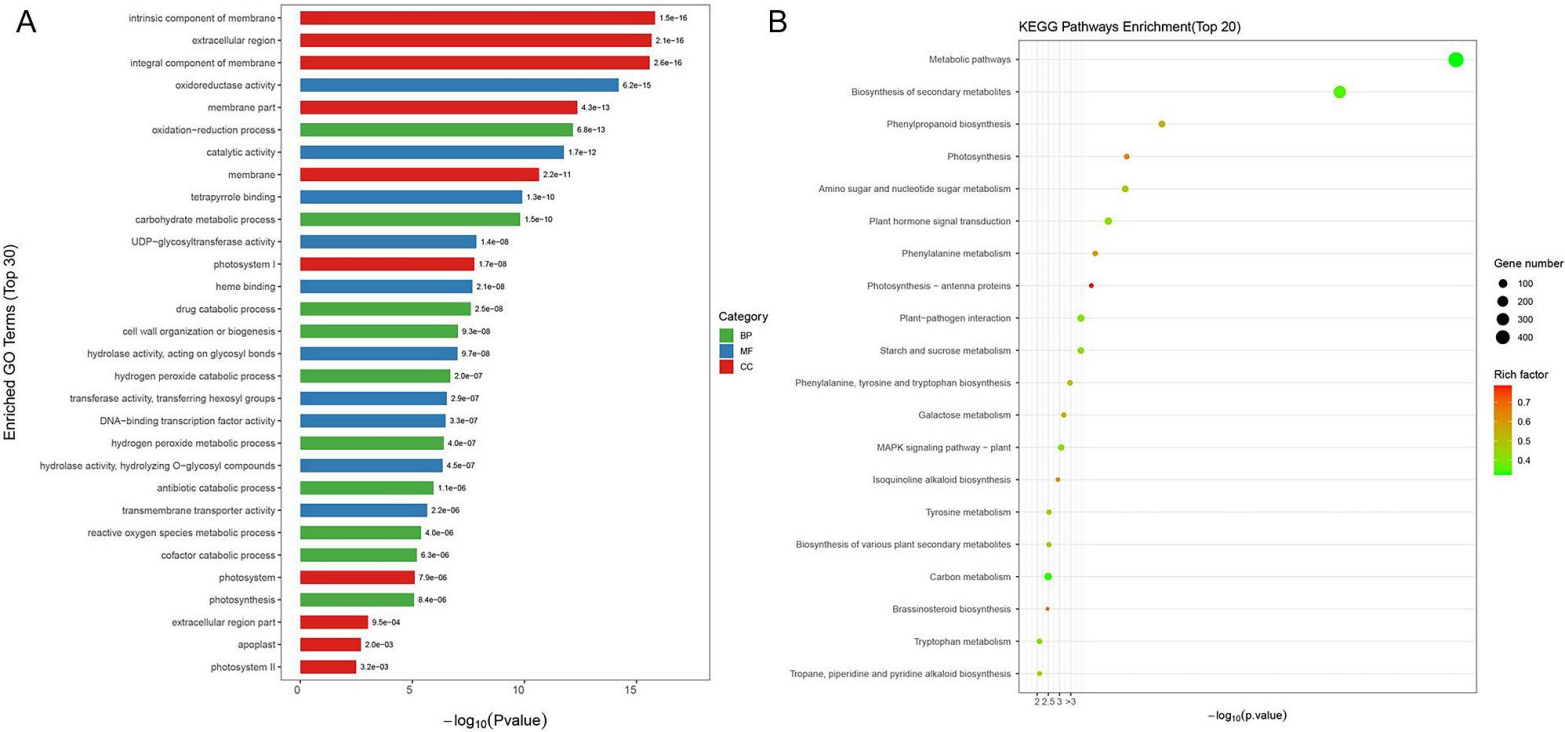
Functional enrichment analysis of genes in the transcriptome of pomelo juice sacs. GO enrichment analysis (**A**), KEGG enrichment analysis (**B**) were shown

The transcriptome data showed that at least 23,247 genes were detected in one sample, of which 5,990 DEGs were identified (Table S1). Gene Ontology (GO) enrichment showed that the entries related to antioxidant enzymes such as oxidoreductase activity, hydrogen peroxide catabolic process and oxidoreductase activity were significantly enriched (Fig. 2A and Table S2). Kyoto Encyclopedia of Genes and Genomes (KEGG) enrichment showed that DEGs was enriched to 22 pathways (*P* < 0.05), among which phenylpropanoid biosynthesis pathway is the most significant one (Fig. 2B and Table S3). Furthermore, the genes encoding antioxidant enzymes including *SOD[Fe]*, *SOD[Mn]*, *CAT*, *GSTs* and *GPX* were also identified (Table S4), which were notably upregulated in granulated juice sacs. In addition, the lignin biosynthesis genes *PALs*, *4CL*, *HCT*, *CCoAMTs*, *CCR*, *CADs* and *PODs* in phenylpropanoid pathway were identified (Table S4), among which *PALs*, *4CL*, *CADs* and *PODs* were significantly upregulated in granulated juice sacs that was consistent with the data of lignin contents (Fig. 1B). Above results indicate that antioxidant enzymes and lignin biosynthesis genes are closely related to the physiological changes in granulated juice sacs of pomelo. To verify the validation of transcriptome data, we selected 10 genes in antioxidant enzyme genes and lignin biosynthesis genes as candidate genes for quantitative real-time PCR (qRT-PCR) validation. The results confirmed the gene expression trends were consistent with DEGs data (Figure S1), showing that our transcriptome result is true and reliable.

### Identification and functional annotation of differentially ubiquitinated proteins (DUPs) in granulated juice sacs of pomelo

**Fig. 3:**
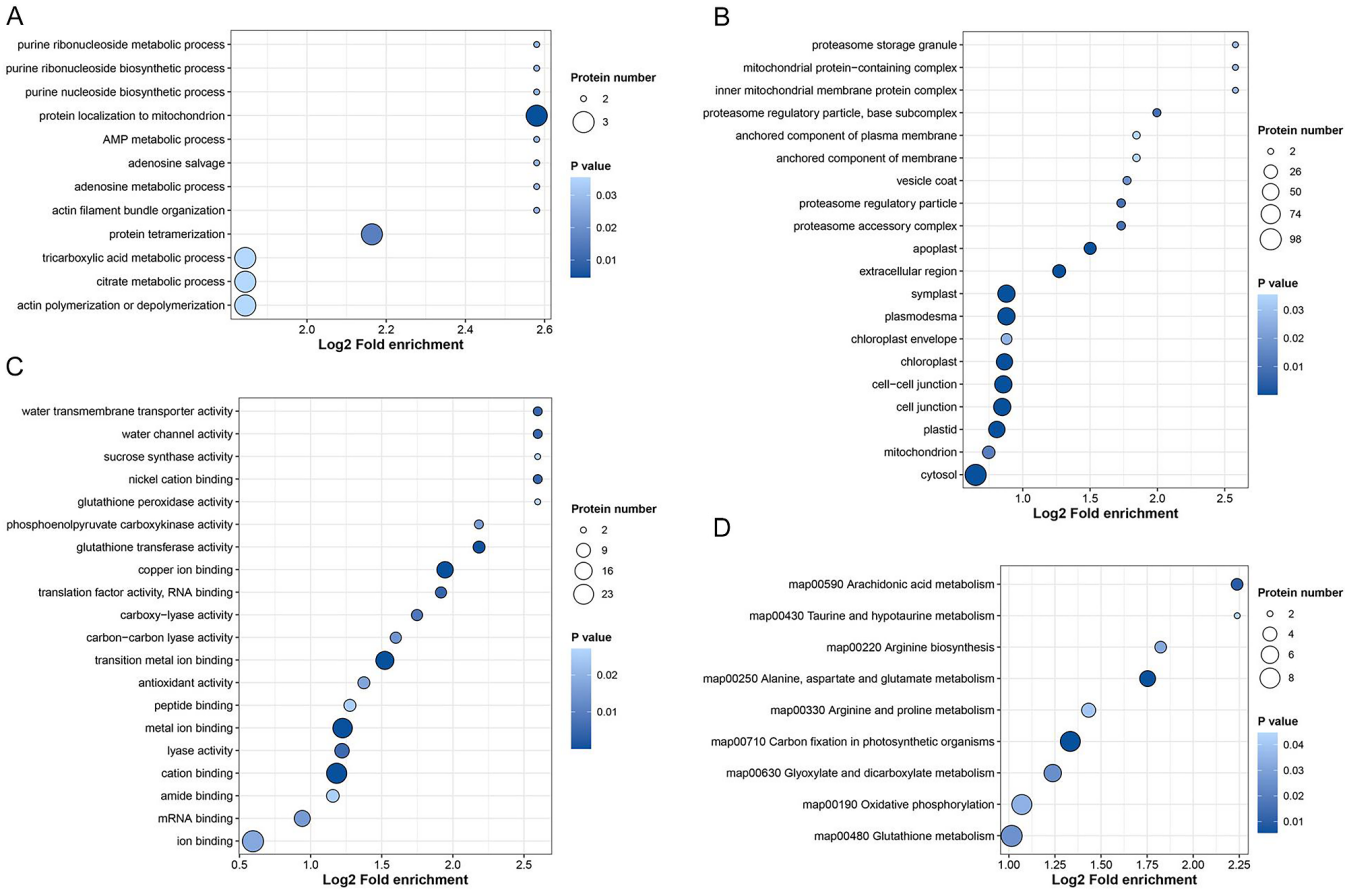
Functional enrichment analysis of proteins in the ubiquitinome of pomelo juice sacs. GO enrichment analysis of biological processes (A), GO enrichment analysis of cellular components (**B**), GO enrichment analysis of molecular functions (**C**), KEGG enrichment analysis (**D**) were shown

Table S5 showed that 505 ubiquitination sites in 314 DUPs were identified in granulated juice sacs. The DUPs were annotated by GO enrichment analysis and divided into three categories including biological processes, cellular components and molecular functions (Fig. 3 and Table S6). In the biological processes, the DUPs were found to be involved in a large number of processes, such as purine ribonucleoside metabolic, purine nucleoside biosynthetic, adenosine metabolic, tricarboxylic acid metabolic and citrate metabolic processes (Fig. 3A). In the cellular components, most DUPs were enriched in the cytosol, symplast, plasmodesma, and cell-cell junction, which showed that ubiquitinated proteins played an important role in these cellular structures (Fig. 3B). In the molecular functions, including the glutathione transferase activity, antioxidant activity and glutathione peroxidase activity were most significantly enriched (Fig. 3C). Furthermore, the pathway related to glutathione metabolism was enriched using the KEGG functional analysis (Fig. 3D and Table S7). Above data suggest that protein lysine ubiquitination is involved in antioxidant enzyme system, which appears to associate with the occurrence of pomelo juice sac granulation.

### Analysis of ubiquitinated antioxidant enzymes and ROS contents in granulated juice sacs of pomelo

**Fig. 4:**
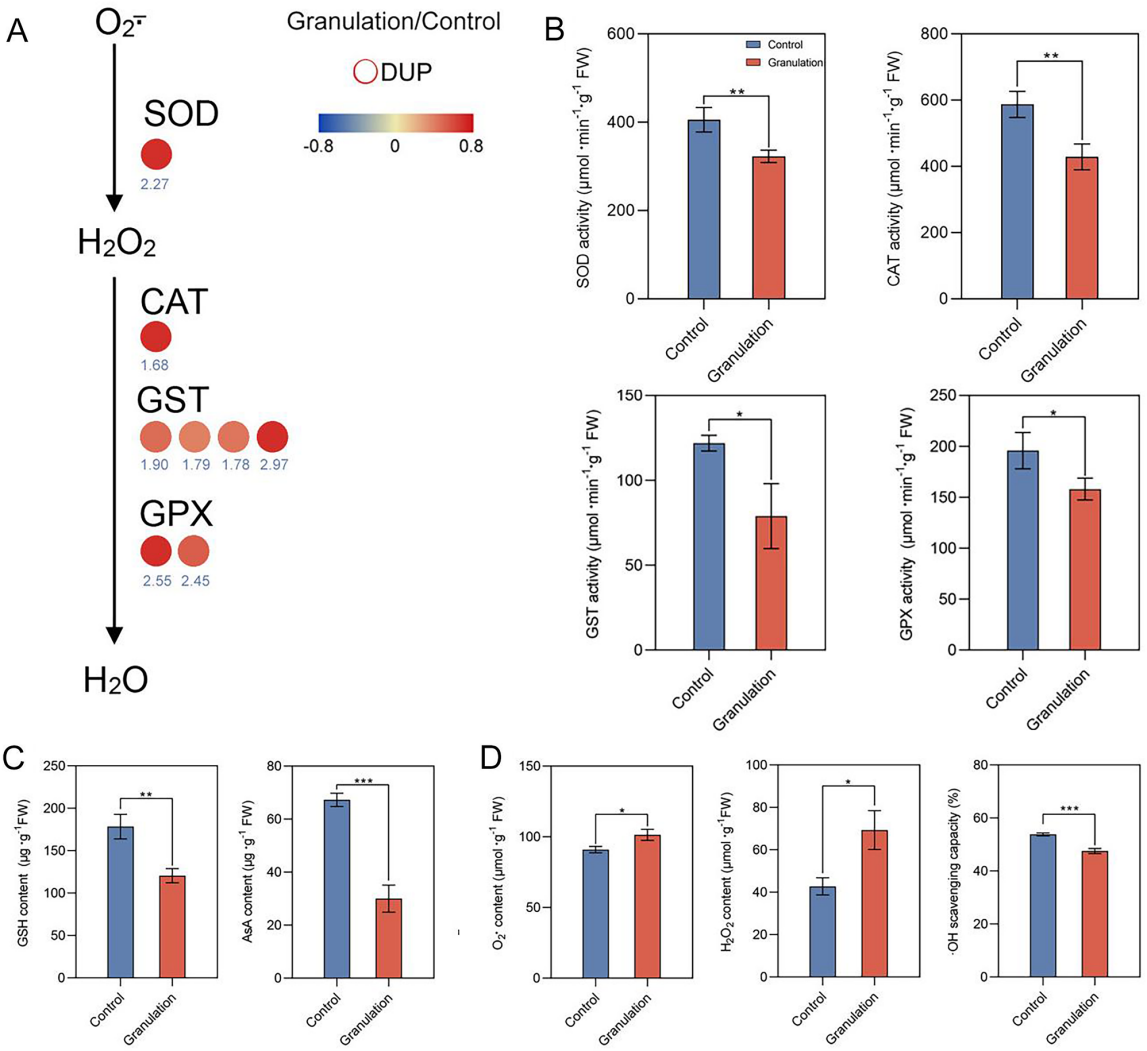
Changes of antioxidant enzyme activities, antioxidant contents and ROS indexes in granulated juice sacs of pomelo. Abundances of ubiquitinated antioxidant enzymes in pomelo juice sacs (**A**), SOD, CAT, GST and GPX activities (**B**), GSH and AsA contents (**C**), O_2_$\stackrel{-}{\bullet }$ and H_2_O_2_ and scavenging capacity of $\bullet$OH (**D**) were shown. Different asterisks represented a significant difference at *p* < 0.05 level by Student’s *t*-test. Control, normal juice sacs; Granulation, granulated juice sacs

Our results showed that antioxidant enzymes, including SOD, CAT, GST and GPX, were significantly increased at ubiquitination level in granulated juice sacs of pomelo (Fig. 4A; Table 1). However, the activities of the four enzymes were decreased in granulated juice sacs of pomelo (Fig. 4B). In addition, two key antioxidants reduced glutathione (GSH) and ascorbic acid (AsA) in the GSH-AsA cycle were determined. The results showed that GSH and AsA contents decreased (Fig. 4C). Furthermore, the results of ROS data demonstrated that superoxide anion (O_2_$\stackrel{-}{\bullet }$ ) and H_2_O_2_ contents increased obviously, and hydroxyl radical ( $\bullet$OH) scavenging capacity decreased in granulated juice sacs (Fig. 4D). Above results indicate that the decreased antioxidant enzyme activities are regulated by the enzyme ubiquitination, which lead to overall decreasing ROS scavenging abilities in granulated juice sacs of pomelo.

**Table 1:** Differentially ubiquitinated antioxidant enzymes in granulated juice sacs of pomelo

Protein accession	Granulation/Control Ratio	*P* value	Regulated Type	KEGG Gene
XP-006471806.1	2.270	0.01869	Up	SOD
XP-006473789.1	1.688	0.03944	Up	CAT
XP-006479908.1	1.901	0.00574	Up	GST
XP-006480546.1	2.976	0.00510	Up	GST
XP-006483268.1	1.798	0.04714	Up	GST
XP-006484982.1	1.786	0.02446	Up	GST
XP-006476598.1	2.456	0.00759	Up	GPX
XP-006494185.1	2.550	0.01281	Up	GPX

*Note* Control: normal juice sacs, Granulation: granulated juice sacs

### Lignin accumulation in response to H_2_O_2_ and DMTU treatments in granulated juice sacs of pomelo

**Fig. 5:**
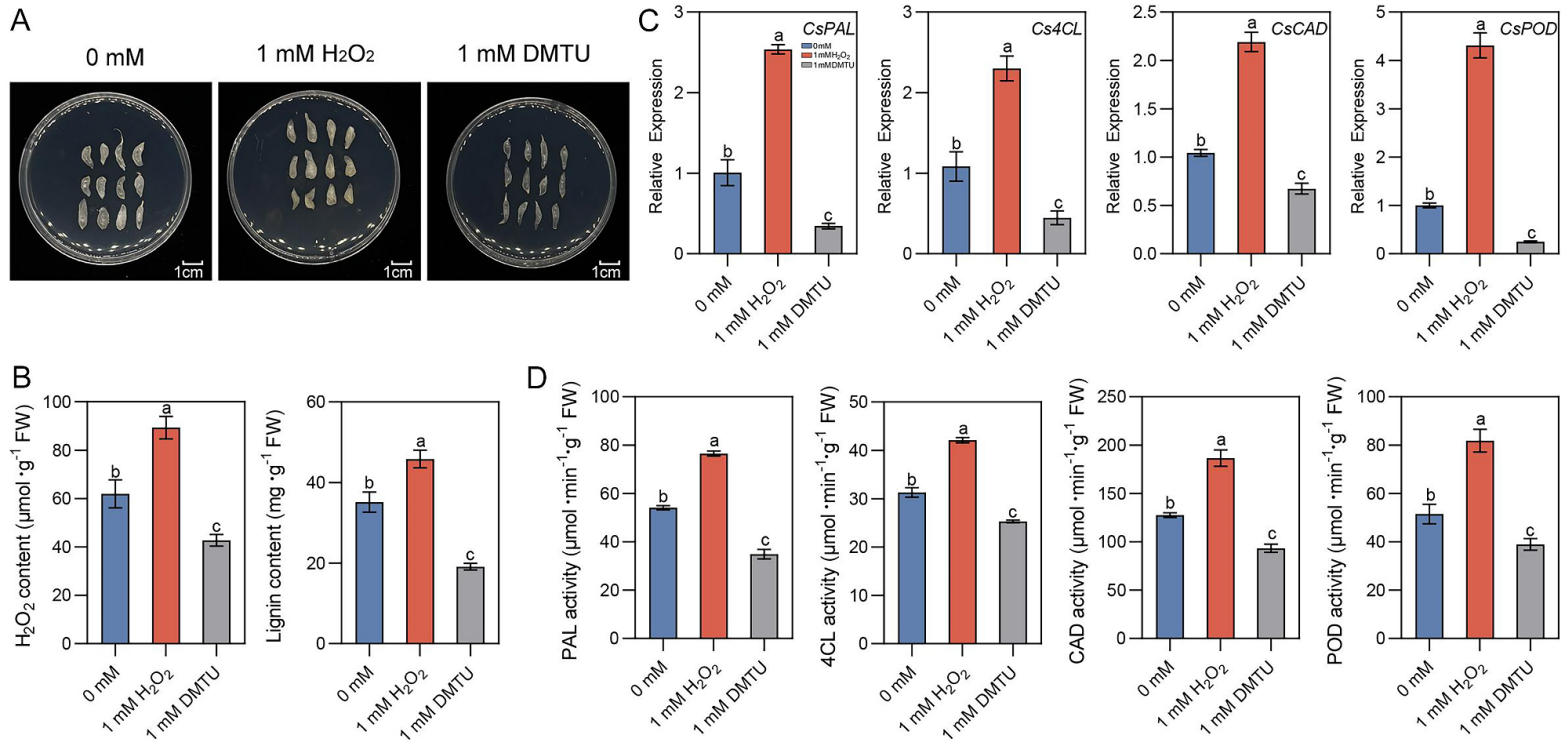
Juice sac granulation can be affected by H_2_O_2_and DMTU treatments through the regulation of lignin contents in pomelo fruits. Phenotype characteristics after H_2_O_2_ and DMTU treatments in pomelo juice sacs (**A**), the contents of H_2_O_2_ and lignin (**B**), the expression levels of lignin biosynthesis pathway genes (**C**), PAL, 4CL, CAD and POD activities (**D**) were shown. Different letters in different treatments represented a significant difference at *p* < 0.05 level by Duncan’s test

We found through ROS data analysis that increased H_2_O_2_ may be correlated with the juice sac granulation of pomelo due to its signaling role. This raises the question of how H_2_O_2_ affects the juice sac granulation. To verify H_2_O_2_ activating the key gene expressions in lignin biosynthesis pathway, the juice sacs of pomelo treated with H_2_O_2_ and DMTU were continuously cultured on the culture medium for 60 days. The degree of juice sac granulation increased obviously after H_2_O_2_ treatment compared with control, and the juice sacs became rough, cloudy and yellow. However, there was no distinct granulation observed in the juice sacs treated with DMTU, and the juice sacs showed transparent (Fig. 5A). In addition, the contents of H_2_O_2_ and lignin in the juice sacs increased after H_2_O_2_ treatment, and inhibited by DMTU treatment (Fig. 5B). qRT-PCR results indicated that the expressions of key genes *CsPAL*, *Cs4CL*, *CsCAD* and *CsPOD* in lignin biosynthesis of the juice sacs treated with H_2_O_2_ were significantly upregulated, while the gene expressions of the juice sacs treated with DMTU were downregulated (Fig. 5C). Similarly, the activities of phenylalanine ammonia-lyase (PAL), 4-coumarate: CoA ligase (4CL), cinnamyl alcohol dehydrogenase (CAD) and peroxidase (POD) in juice sacs treated with H_2_O_2_ increased, while the enzyme activities in juice sacs treated with DMTU decreased (Fig. 5D). These results confirm that H_2_O_2_ can induce the increases of the relative enzyme activities by up-regulating the expression of the key genes in lignin synthesis pathway, promoting the accumulation of lignin and finally leading to the pomelo juice sac granulation.

## Discussion

In recent years, juice sac granulation, an important physiological disorder affecting the quality of citrus fruits, has been wildly concerned. Previous studies reported that the contents of cell wall components in granulated juice sacs had been changed, in which lignin was specifically accumulated [10, 18]. Lignin is a phenolic polymer deposited in plant secondary cell walls. The change of lignin contents is connected with the expressions of the genes related to lignin biosynthesis pathway such as *PAL*, *C4H*, *4CL*, *CCR*, *CAD* and *POD*, which plays a vital role in the process of lignin biosynthesis in fruits [2, 19]. In the present study, we identified the genes of *PALs*, *4CL*, *HCT*, *COMTs*, *CCoAMTs*, *CCR*, *CADs* and *PODs* in lignin biosynthesis pathway, and the expression levels of the most genes were significantly upregulated in granulated juice sacs. These results were consistent with the change of lignin content in this study, indicating that the genes regulate the lignin biosynthesis in granulated juice sacs of pomelo. Therefore, lignin accumulation is an important cause of juice sac granulation, which declines pomelo fruit quality [20].

To our knowledge, investigations of ubiquitinome in response to physiological disorders have not been reported in any fruit plants. Protein ubiquitination has been shown to play important roles in diverse biological processes including gene expression, protein activity, salt and drought stress, carotenoid and anthocyanin metabolism in plants [21, 22]. In the present study, 5,361 ubiquitination sites were identified on 1,990 proteins in pomelo juice sacs, and the ubiquitination sites and proteins in pomelo juice sacs are more than those of most reported plants such as tea and maize [23–25], indicating an essential role of ubiquitinated proteins in pomelo juice sacs. Furthermore, 314 proteins in the granulated juice sacs were defined as DUPs, 96.49% of which were upregulated, indicating that these upregulated DUPs might be related to the granulation of juice sacs. Moreover, GO and KEGG analyses showed that the upregulated DUPs such as antioxidant enzymes were significantly enriched in the glutathione transferase activity, antioxidant activity, glutathione peroxidase activity and glutathione metabolism, implying an essential role in the granulated juice sacs of pomelo.

Increasing evidences show that antioxidant enzymes play crucial role in the regulation of ROS metabolism in plant cells. The enzymes are essential for scavenging ROS, maintaining the dynamic balance of ROS in the cells [26]. Antioxidants such as AsA and GSH are also effective in preventing ROS generation and accumulation in plants [27]. We notice that numerous protein/enzyme activities can be negatively regulated by ubiquitination. For example, increasing the level of ubiquitination of Nrf2 inhibits the activity of ARE, leading to the accumulation of intracellular ROS [28]. Many receptor tyrosine kinases (RTKs) are negatively regulated by ubiquitination [29]. In the present study, we found that the genes expression levels of *SOD*, *CAT, GST* and *GPX* mined from transcriptome were significantly upregulated, and the relative enzymes screened from ubiquitinome were obviously increased in abundance while the enzyme activity were significantly reduced in granulated juice sacs. These results suggest that the enzyme activities are negatively affected by ubiquitination modification, resulting in a decrement of the enzyme activities in granulated juice sacs of pomelo. However, there is no instance where the ubiquitination levels of antioxidant enzymes have been directly related to the enzyme activities, which needs further study. Furthermore, we found that antioxidant GSH and AsA contents decreased in the granulated juice sacs. Additionally, O_2_$\stackrel{-}{\bullet }$ production rate and H_2_O_2_ content increased, and $\bullet$OH scavenging capacity were also found to be decreased in the granulated juice sacs. Taken together, these results suggest that the total ROS scavenging ability is reduced, leading to the increase of ROS in the granulated juice sacs. Although ROS are highly toxic substances, many investigations have verified that ROS such as H_2_O_2_ and O_2_$\stackrel{-}{\bullet }$ are vital signaling messengers and playing an essencial role in controlling a variety of biological processes within plant cells [30]. These results suggest that ROS levels are increased in the granulated juice sacs of pomelo due to the decreased activities of antioxidant enzymes, which are regulated by the enzyme ubiquitination.

Lignin is a phenolic polymer synthesized within cell walls of plants [31]. Peroxidases and/or laccases located in cell walls activate lignin monomer to form the lignin polymers [30]. In addition, many previous studies reported that ROS signaling might be important in the plant lignification [32, 33]. H_2_O_2_ is fairly stable compared to other ROS in plant cells, and is thus considered as the dominant ROS involved in both cellular signaling and the lignification process [34]. Previous studies indicate that H_2_O_2_ is necessary for lignin biosynthesis in postharvest bamboo shoots. The endogenous H_2_O_2_ acts as a crucial signaling molecule activating the enzyme activities of PAL, C4H, and 4CL, which promotes the lignin biosynthesis [35]. Moreover, the *PuPOD2* and *PuLAC2* response to H_2_O_2_ is modulated at the gene transcription level, which induces the expressions of these two genes regulating lignin biosynthesis in pear calli [36]. Similarly, in the present study, the endogenous H_2_O_2_ content was found to be accumulated in the granulated juice sacs of pomelo, and the key gene expressions levels of *CsPAL*, *Cs4CL*, *CsCAD* and *CsPOD* in lignin biosynthesis pathway was remarkably higher compared with normal juice sacs. Additionally, we found that the change trend of H_2_O_2_ contents was consistent with lignin accumulation in pomelo juice sacs. Notably, the signaling role of H_2_O_2_ on regulating these gene expressions leaded to lignin accumulation in the granulated juice sacs, which was verified using tissue culture by adding exogenous H_2_O_2_ and DMTU. This result indicates that the expressions of lignin synthesis genes *CsPAL*, *Cs4CL*, *CsCAD* and Cs*POD* are upregulated by H_2_O_2_ signalling, and the activities of the relative enzymes are increased, which contributes to lignin accumulation in the granulated juice sacs of pomelo fruits. As an inhibitor of H_2_O_2_ production, DMTU can reduce granulation process in the juice sacs. This result was consistent with the previous report [37]. Taken together, we suggest that increased H_2_O_2_ activates the gene expressions and enzyme activities in lignin synthesis pathway, which eventually leads to lignification in the granulated juice sacs of pomelo.

## Conclusions

The present study is the first report focusing on the molecular basis at the level of transcriptome and ubiquitinome regarding to the juice sac granulation of pomelo. We found there were two ways of molecular regulation on the lignification in the granulated juice sacs of pomelo. Firstly, the lignification is modulated by upregulated gene expressions in lignin biosynthesis pathway, which leads to lignin accumulation and induces the juice sac granulation. Secondly, the lignification is also regulated by deceased activities of antioxidant enzymes due to ubiquitination modification, which results in the increase of endogenous H_2_O_2_ content in the granulated juice sacs. H_2_O_2_ might act as a signaling molecule to activate the gene expressions and enzyme activities in the lignin biosynthesis pathway, promoting lignin accumulation and inducing juice sac granulation of pomelo. These results suggest that antioxidant enzyme ubiquitination may be associated with juice sac granulation of the fruit. Our findings provide new leads for further pomelo production in mitigating juice sac granulation and improving fruit quality.

## Materials and methods

### Plant materials

Guanxi pomelo (*Citrus grandis* L. Osbeck) fruits were collected at maturity (about 215 days after anthesis) in an orchard of Pinghe County, Fujian Province, China. The juice sacs were divided into control group (normal juice sacs) and granulation group (granulated juice sacs). Juice sacs from the samples of control and granulation in each fruit were collected and frozen using liquid nitrogen, and stored at -80 °C for RNA extraction, transcriptome sequencing, ubiquitinome analysis, lignin content determination and enzyme activity assay. Three biological replicates were used for all the above experiments.

### Lignin content determination

As described by Shi et al. [38], 8 g juice sacs were ground to powder and homogenized in 15 ml wash buffer (100mM K_2_HPO_4_/KH_2_PO_4_, 0.5% Triton X-100, 0.5% PVP-K30; pH 7.8), and then washed on a shaker for 15 min at room temperature. Subsequently, the mixture was centrifuged at 15,000 g for 15 min at 4 °C and the supernatant was discarded (Eppendorf, Germany). The washing process was repeated three times. Then the precipitates were washed three times with ddH_2_O and dried in a vacuum at 60 °C overnight. The dried powder was resuspended in 2 ml of 1.0 M NaOH and centrifuged at 15,000 g for 20 min at 4 °C. 0.1 ml HCl was added to the supernatant (500 µl), incubated to precipitate the lignin thioglycolic acid and centrifugated at 15,000 g for 15 min at 4 °C. The precipitate was then dissolved in 1.0 M NaOH (1:100 ml v/v) and was measured at 280 nm by ultraviolet spectrophotometry (U-T3C, China).

### RNA extraction, cDNA library construction and RNA sequencing

Total RNA was extracted according to the manufacturer’s instructions (TianGen, China) from pomelo juice sacs. The cDNA library construction and RNA sequencing were performed by APTBIO (Shanghai, China). To obtain a clear raw read, the Illumina HiSeq sequencing platform was used to sequence the libraries. After being filtered by HISAT2, all of the clean reads have been mapped to the *Citrus sinensis* reference genome. The featureCounts software is then used to calculate expression values of FPKM for the genes in each sample.

### Quantitative real-time PCR

As the manufacturer’s instructions (TianGen, China), total RNA was extracted from pomelo juice sacs, and the RNA was synthesized into cDNA by the commercial kit (Vazyme, China). The qRT-PCR was carried out using qRT-PCR kit (Vazyme, China) with a light cycler 96 system (Roche, Switzerland). The actin gene has been used as an internal standard, and the 2^−ΔΔCt^ method has been applied for qRT-PCR data analysis [39]. Table S8 listed all primer sequences. For each sample, a total of three separate biological replications were carried out.

### Protein extraction

Proteins of pomelo juice sacs were extracted according to previous descriptions [40]. Pomelo juice sacs (0.5 g) were ground to powder using liquid nitrogen and resuspended in lysis buffer (10 mM dithiothreitol, 1% protease inhibitor cocktail, 50 µM PR-619, 3 µM TSA, 50 mM NAM), and then sonicated on ice for 3 times with a high-intensity ultrasonic processor (Scientz, China). Subsequently, an equal volume of Tris-saturated phenol (pH 8.0) was added, and the supernatant was collected by centrifugation at 20,000 g for 15 min at 4 °C. Next, the supernatant was precipitated overnight by adding 0.1 M ammonium acetate/methanol. Then, the precipitates were washed 3 times with cold acetone, then discarded the supernatant after centrifugation at 15,000 g for 20 min at 4 °C. The precipitates were resuspended in urea and the protein concentration was determined using the BCA kit (Beyotime, China) according to manufacturer’s instructions.

### Trypsin digestion of proteins

Tryptic digestion for the protein samples of pomelo juice sacs was performed as described previously [41]. The protein solution was reduced with 5 mM dithiothreitol (DTT) for 30 min at 56 °C and alkylated with 11 mM iodoacetamide (IAM) for 15 min at room temperature in darkness. Then 200 mM TEAB was added to dilute the protein. Trypsin was added, and the mass ratio of trypsin to protein was 1:50 and 1:100, respectively. The first digestion was done overnight, and the second one for 4 h. Three biological replicates were performed.

### Affinity enrichment of ubiquitinated proteins

Ubiquitinated proteins of pomelo juice sacs were enriched as described previously [41]. For enrichment of lysine-ubiquitinated peptides, the tryptic peptides were dissolved in IP buffer (100 mM NaCl, 1 mM EDTA, 50 mM Tris-HCl, 0.5% NP-40, pH 8.0) and incubated with prewashed anti-K-ε-GG antibody beads (Jingjie, China) overnight at 4 °C with moderate oscillation. Subsequently, the beads were washed with IP buffer 4 times and with ddH_2_O twice. The beads were eluted three times with 0.1% trifluoroacetic acid (w/v). The eluted fractions were collected and dried in a vacuum overnight. Following the manufacturer’s instructions, the resulting peptides were cleaned using C18 ZipTips (Millipore).

### LC-MS/MS analysis

For LC-MS/MS analysis, ubiquitinated peptides of pomelo juice sacs were conducted as previously studied [42]. The ubiquitinated peptides dissolved into solvent A (0.1% formic acid, 2% acetonitrile) and the peptides were separated by gradient in 8 − 80% solvent B (0.1% formic acid, 90% acetonitrile). The peptides were then separated by an EASYnLC 1200 UPLC system (Thermo Scientific) and injected into the NSI Ion Source for ionisation and then analyzed by mass spectrometry on an Orbitrap Exploris™ 480 (Thermo Fisher Scientific).

### Database search and bioinformatic analysis

The levels of gene expressions were calculated by FPKM (Fragments Per Kilobase of exon model per Million mapped fragments). Using a threshold of *p* < 0.05 and |fold-change| >1.5 or |fold-change| < 1/1.5, the DEGs in pomelo juice sacs were identified. The ubiquitinated proteins and sites have been identified using Andromeda search engine on Max Quant (v.1.5.2.8). The obtained MS/MS data was searched against the *Citrus sinensis* uniprot database sequences concatenated with the reverse decoy database. The DUPs or modified Kub sites in pomelo juice sacs were identified with a threshold of *p* < 0.05 and |fold-change| >1.5 or |fold-change| < 1/1.5. GO and KEGG public databases were used to annotate all the transcripts and ubiquitinated proteins.

### Determination of the indexes related to ROS metabolism

By manufacturer’s instructions, the antioxidant enzyme activities were measured with SOD, CAT, GST and GPX commercial assay kits (Solarbio, China), and the absorbance were determined at 560 nm, 240 nm, 412 nm and 340 nm, respectively.

The antioxidant contents were detected using GSH and AsA commercial assay kits (Solarbio, China), and the absorbance was determined at 265 nm and 412 nm, respectively.

The O_2_$\stackrel{-}{\bullet }$ production rate, H_2_O_2_ content, and hydroxyl radical $\bullet$OH scavenging capacity were detected using commercial assay kits (Solarbio, China), and the absorbance was determined at 530 nm, 415 nm and 536 nm, respectively.

### Pomelo juice sacs with H_2_O_2_ and DMTU treatments

The juice sacs of the Guanxi pomelo were collected at 150 days after anthesis in 2023 and cultured on murashige and skoog medium with concentration of 100 µ mol L^− 1^ H_2_O_2_ (Xilongs, China) and DMTU (a scavenger of H_2_O_2_; Macklin, China). After being cultured for 60 days on the media, the samples were used to evaluate H_2_O_2_ and lignin contents, lignin biosynthesis gene expressions and enzyme activities.

### Determination of enzyme activities related to lignin biosynthesis

PAL, 4CL, CAD and POD activities were measured by commercial assay kits (Solarbio, China), and the absorbance was determined at 290 nm, 333 nm, 340 nm and 470 nm, respectively.

### Statistical analysis

Gene expression and ubiquitinated protein profiles were processed with Excel, SPSS 26.0 and GraphPad Prism 8.0. Duncan’s multiple comparison test or Student’s *t*-test is used to calculate the statistical significance of the difference, and *P* < 0.05 is considered to be significant.

### Electronic supplementary material

Below is the link to the electronic supplementary material.

Supplementary Material 1

Supplementary Material 2

## Data Availability

Sequence data that support the findings of this study have been deposited in the European Nucleotide Archive with the primary accession code PRJNA1077242.
