# Supplementary material for: A multilevel investigation to reveal the regulatory mechanism of lignin accumulation in juice sac granulation of pomelo
Source: BMC Plant Biol. 2024 May 11;24:390. doi: 10.1186/s12870-024-05095-4 (PMC11088010; doi:10.1186/s12870-024-05095-4)
Supplement: Supplementary file 1 — Supplementary Material 1 [file 12870_2024_5095_MOESM1_ESM.docx]

**Additional file 1:**

**
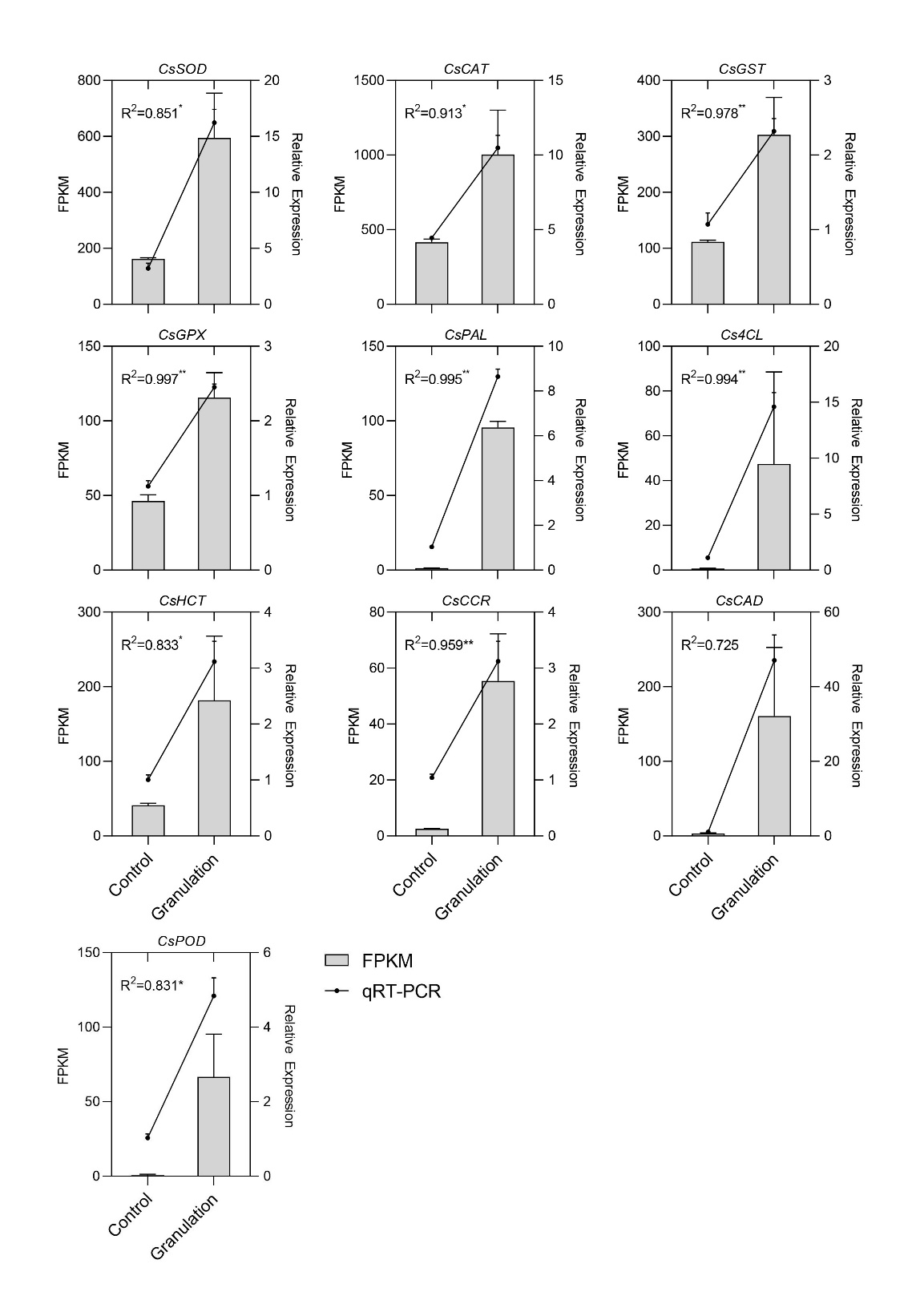
Figure S1.** The expression level of 10 DEGs related to antioxidant enzyme and lignin biosynthesis was based on the FPKM value. FPKM values were the means of three replications.
